# Scaling-law mechanical marker for liver fibrosis diagnosis and drug screening through machine learning

**DOI:** 10.3389/fbioe.2024.1404508

**Published:** 2024-07-16

**Authors:** Honghao Zhang, Jiu-Tao Hang, Zhuo Chang, Suihuai Yu, Hui Yang, Guang-Kui Xu

**Affiliations:** ^1^ School of Mechanical Engineering, Northwestern Polytechnical University, Xi’an, China; ^2^ Department of Engineering Mechanics, SVL, School of Aerospace Engineering, Xi’an Jiaotong University, Xi’an, China; ^3^ School of Life Sciences, Northwestern Polytechnical University, Xi’an, China

**Keywords:** cell mechanics, viscoelastic, machine learning, rheology, liver diagnosis

## Abstract

Studies of cell and tissue mechanics have shown that significant changes in cell and tissue mechanics during lesions and cancers are observed, which provides new mechanical markers for disease diagnosis based on machine learning. However, due to the lack of effective mechanic markers, only elastic modulus and iconographic features are currently used as markers, which greatly limits the application of cell and tissue mechanics in disease diagnosis. Here, we develop a liver pathological state classifier through a support vector machine method, based on high dimensional viscoelastic mechanical data. Accurate diagnosis and grading of hepatic fibrosis facilitates early detection and treatment and may provide an assessment tool for drug development. To this end, we used the viscoelastic parameters obtained from the analysis of creep responses of liver tissues by a self-similar hierarchical model and built a liver state classifier based on machine learning. Using this classifier, we implemented a fast classification of healthy, diseased, and mesenchymal stem cells (MSCs)-treated fibrotic live tissues, and our results showed that the classification accuracy of healthy and diseased livers can reach 0.99, and the classification accuracy of the three liver tissues mixed also reached 0.82. Finally, we provide screening methods for markers in the context of massive data as well as high-dimensional viscoelastic variables based on feature ablation for drug development and accurate grading of liver fibrosis. We propose a novel classifier that uses the dynamical mechanical variables as input markers, which can identify healthy, diseased, and post-treatment liver tissues.

## Introduction

Liver cirrhosis and cancer are serious liver diseases with high mortality rates due to their irreversibility (Tapper and Loomba, 2018; Agarwal et al., 2019), whereas liver fibrosis is the early stage of them (Friedman, 2010; Seitz et al., 2018; Stefan et al., 2019) and could be reversed by rational medication (Li et al., 2018; Salarian et al., 2019). To date, early diagnosis and quantification of the extent of liver fibrosis are of great clinical value for timely intervention and reversing the development of liver fibrosis (Friedman, 2010; Zhao et al., 2017; Tapper and Loomba, 2018; Balachandran et al., 2022). The current gold standard for diagnosing liver disease is liver biopsy, which relies on the pathological examination of tissue samples obtained through invasive puncture. However, the invasive nature of biopsies significantly diminishes the patient experience and can potentially lead to complications (Eskew et al., 1997; Yasufuku and Fujisawa, 2007; Veronesi et al., 2010). In contrast, ultrasound has gained widespread applications as the preferred method for clinically screening liver diseases due to its radiation-free nature, cost-effectiveness, convenience, and invaluable real-time imaging capabilities (Bamber et al., 2013; Tapper and Loomba, 2018).

Currently, the primary diagnostic methods for the degree of liver fibrosis are semi-quantitative methods (Sun et al., 2017; Xu et al., 2021), such as liver examinations based on clinical, biochemical, and imaging methods. Therefore, there is a lack of a satisfactory method to accurately determine the degree of hepatic fibrosis. Liver pathology is a complex process characterized by various features at different stages. Utilizing a combination of diagnostic methods is advantageous compared to relying on a single biomarker, as it offers supplementary insights into the condition of the liver. It is well known that the mechanical properties of cells and tissues are closely related to their pathological states (Suresh, 2007; Grant and Twigg, 2013; Rigato et al., 2017; Mandal et al., 2019; Staunton et al., 2019; Guimarães et al., 2020). Utilizing elastography, it is possible to derive the modulus of elasticity of liver tissue and assess the grading of lesions based on their mechanical properties. Many experiments showed that the elastic stiffness is positively correlated with the degree of liver fibrosis (Ziol et al., 2005; Yin et al., 2007). Recent studies (Lei et al., 2017; Lewindon et al., 2019; Xue et al., 2020) have demonstrated that the combination of biochemical and mechanical parameters, along with imaging and ultrasound techniques, exhibits a markedly enhanced diagnostic efficacy for liver lesions compared to individual parameters alone. In addition, SVM has been successfully applied to classify cancerous and normal cells, yielding promising results (Wang et al., 2021). Linking mechanical properties to pathological states provides a novel precise and robust diagnostic marker for diagnosis (Staunton et al., 2019; Wang et al., 2021). However, soft biological tissues are not elastic materials, yet similar to living cells (Fabry et al., 2001; Dimitrije et al., 2004; Smith et al., 2005; Hoffman et al., 2006; Koenderink et al., 2009; Rigato et al., 2017; Hu et al., 2019), they are a viscoelastic material that exhibits a fascinating scaling-law creep response (Liu and Bilston, 2000; Chaudhuri et al., 2016; Chaudhuri et al., 2020). For liver tissues, scaling-law response is also observed in experiments (Chang et al., 2023). It puts doubts as to whether a single value of elastic modulus sufficiently discriminates the pathological stage of liver fibrosis. The correlation between the viscoelastic mechanical properties of liver tissue and liver lesions is currently unexplored. Therefore, quantifying the viscoelastic mechanical properties during liver fibrosis development can provide additional mechanical markers to grade the degree of liver fibrosis and to evaluate the effect of drug treatment, which further improves the precision of diagnosis. However, the high-dimensional mechanical data generated by viscoelastic characterization poses new challenges for evaluating the mechanics of liver fibrosis. The application of machine learning for medical diagnosis in imaging (Kononenko, 2001; Komura and Ishikawa, 2019; Soelistyo et al., 2022) provides us with a viable means to deal with such high-dimensional data.

In this study, we obtained the creep responses of mouse liver tissue sections by atomic force microscopy (AFM). Then, we characterized their creep responses using the self-similar hierarchical model and then acquired high-dimensional viscoelastic mechanical data of healthy, diseased, and MSCs-treated fibrotic liver tissues. Based on a supervised machine learning algorithm, the support vector machine (SVM) method is applied to discover useful mechanical markers, exploiting the hidden associations between viscoelastic parameters with liver pathological states. The combination of high-dimensional viscoelastic mechanical data and machine learning algorithm trained a liver pathological states classifier and the rest of the untrained data was used to test this classifier. We showed that the classifier could achieve 99% accuracy for healthy and diseased livers, 86% accuracy for healthy and MSCs-treated fibrotic livers, and 82% accuracy for a mixture of the three livers together using the viscoelastic mechanical parameters as the input markers.

## Methods

Sections of the liver tissues of the mouse were used as the experimental object to acquire a more accurate viscoelastic mechanical response. The mouse liver tissues in the test were divided into three groups: healthy, diseased, and MSCs-treated fibrotic livers. The staging of liver biopsies through the fibrosis scoring systems, such as Batts and Ludwig or Metavir, is deemed most appropriate. Currently, achieving precise modulation for accurate staging of liver lesions during mouse culture remains challenging. Thus, we categorized the mice into three groups: healthy, diseased, and MSCs-treated fibrotic livers. During the mouse culture process, we implemented a relatively prolonged culture period to induce the development of noticeable lesions, with therapeutic drug injection serving as an intermediary state between the healthy and diseased states. C57BL/6 Mice were randomly assigned to three groups. The control group consisted of healthy, wildtype mice that did not receive any injections of MSCs therapy. Liver fibrosis was induced in the other two groups (diseased and MSCs-treated fibrotic groups, *n* = 2) by intraperitoneal injections of therapeutic drug (1 μL/g) for 7 weeks. At the end of the sixth week, half of the mice received a single intravenous infusion of 2 x105 MSCs. These mice constituted the MSCs-treated fibrotic groups. In the initial step, mouse liver extraction was performed, with particular attention given to isolating the tissues surrounding the portal veins, which connect the left lobe to the rest of the liver tissue. Subsequently, the liver tissues were immediately frozen at - 80°C and cryo-sectioned to a nominal thickness of 15 µm using a Leica CM1850 cryostat (Leica Microsystems (United Kingdom) Ltd., Milton Keynes) and adhered to glass coverslips for future research. Afterward, the dynamical creep indentation test was performed on cells after conducting Masson’s trichrome staining, Sirius Red staining, and aspartate aminotransferase (AST) assay. To mitigate the effects of local remodeling events on the tissue structure under investigation, measurements were carried out at multiple locations separated by a significant distance (i.e., > 50 μm). To reduce the influence of stiff collagen on tissue during characterization, dynamical and static indentation experiments were intentionally conducted away from the portal zones.

**FIGURE 1:**
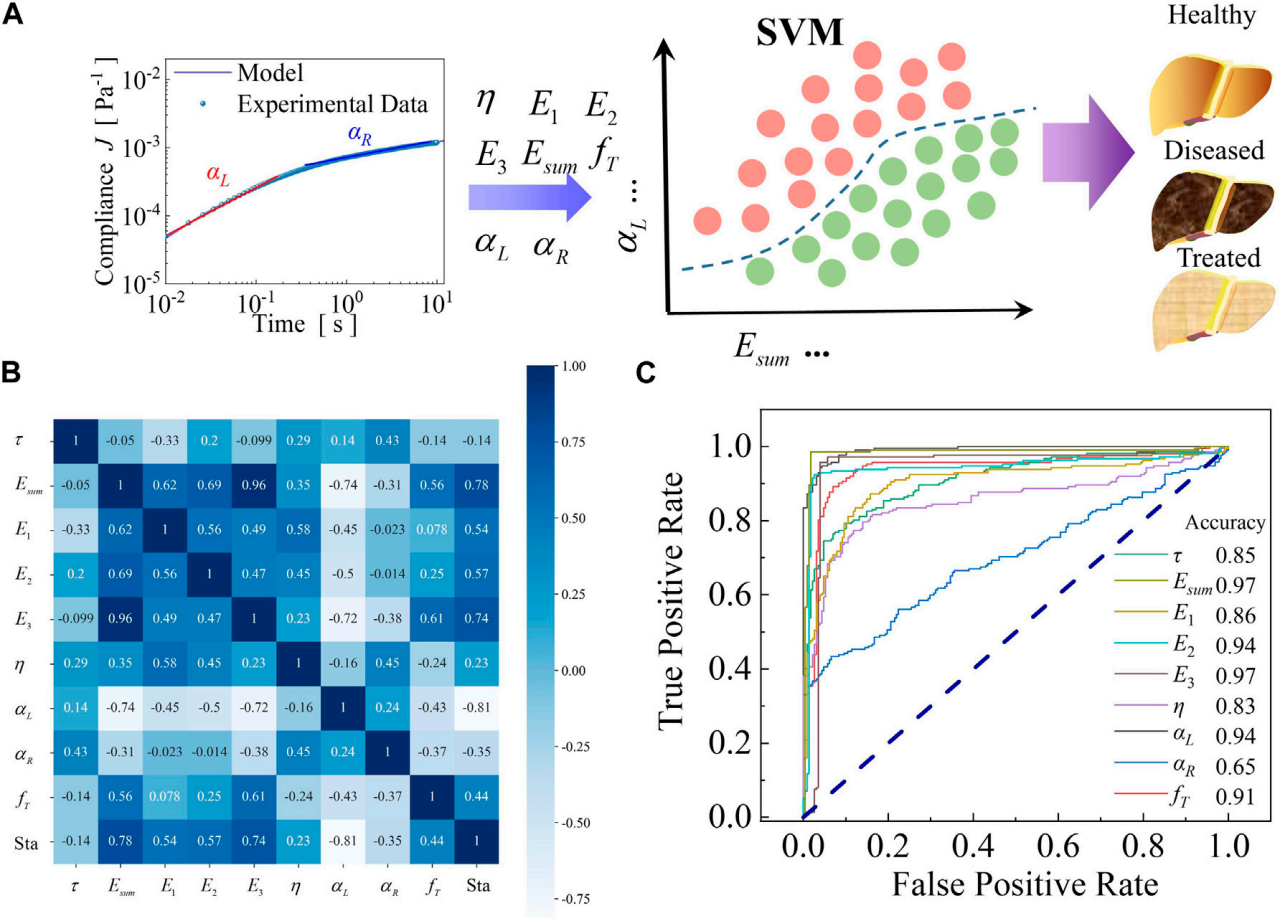
Performance assessment of single viscoelastic marker in the test data group of healthy and diseased livers. **(A)** Overview of machine learning-based liver states classification and diagnosis using viscoelastic mechanical parameters. $E_{1}$ , $E_{2}$ , $E_{3}$ and η can be obtained by performing creep testing through AFM and then characterizing them using the self-similar hierarchical model, while $E_{sum}$ and $f_{T}$ can be obtained by model parameter operations. The double power-law exponents ( $\alpha_{L}$ and $\alpha_{R}$ ) at different time scales can be obtained by the double power-law characterization of the creep compliance. The viscoelastic mechanical data set from the characterization of liver tissues is used to train the machine learning models for the classification of liver states. **(B)** The heat map of the Pearson correlation coefficient of viscoelastic variables between tissue status and viscoelastic variables of healthy and diseased livers. **(C)** Receiver operating characteristics (ROCs) of the classifier with each viscoelastic variable as a single mechanical marker input.

To obtain the viscoelastic mechanical properties of liver samples, the creep responses of the liver tissues were obtained by applying step stress to the samples by AFM with a customized spherical probe (diameter = 20 μm) and holding for 10 s. Each creep compliance indentation test was performed randomly on tissue sections with 100 μm spacing between two locations. Due to the relatively large sample area (∼1 cm^2^), each test performed by the micron-size spherical probe is regarded as a single mechanical measurement on 1 mouse. Each group received 800 measurements. Then, the viscosity ( η ) of cytoplasm, the elastic moduli of cytoplasm ( $E_{1}$ ), cytoskeleton fiber ( $E_{2}$ ), and whole cell structural network ( $E_{3}$ ), and the transition frequency ( $f_{T}$ ) can be acquired by characterizing the creep responses of liver tissues (Figure 1A) based on our previous self-similar hierarchical model (Hang et al., 2021; Hang et al., 2022), which provides a richer and physically meaningful description of the rheological behavior of biological tissues. The model can well fit the creep responses of cells and tissues, and more details about the model fitting can be found in our previous work (Hang et al., 2021; Hang et al., 2022). The R^2^ values of creep compliance for the three liver tissues fitted using the self-similar hierarchical model are all above 0.9. The spherical indenter was used to apply step stress to the liver tissue sections, and the creep compliance expression can be obtained by the Hertz model (Lin and Horkay, 2008):

J=4Rδ323F1−ν2, (1)

where *R* and δ are the radius and indentation depth of the spherical indenter, *F* is the step force, and ν is the Poisson’s ratio with a value of 0.5 (i.e., incompressible). Experiments showed that the creep compliances (Eq. 1) of all three groups exhibit a universal two-stage scaling-law viscoelastic rheology (Chang et al., 2023). Typical creep curves of liver tissue showed that the power-law exponent of creep compliance is not constant with increasing loading time, reaching between 0.5 and 1.0 at small time scales ( $\alpha_{L}$ ) and stabilizing around 0.2 at larger timescales ( $\alpha_{R}$ ), which corresponds to the double power-law viscoelastic behavior of the complex modulus in the frequency domain (Rigato et al., 2017; Hurst et al., 2021). The double power-law viscoelastic model provides novel viscoelastic parameters as mechanical markers for liver fibrosis. SVM is well-suited for the analysis of high-dimensional data sets comprising numerous features, due to its ability to map data into a high-dimensional space. In such scenarios, alternative classification algorithms often encounter dimensionality catastrophes, whereas SVMs efficiently handle the complexities of high-dimensional data (Dumais et al., 1998; Cristianini and Shawe-Taylor, 1999; Evgeniou et al., 2015). Since the individual viscoelastic parameters are not stand-alone and the training data are linearly inseparable, a nonlinear SVM classifier is developed by kernel method and soft interval maximization. The objective function for classification optimization is

$$\min\left(\frac{1}{2}{\left\|w\right\|}^{2}\right)+C\sum_{i=1}^{n}\xi_{i},$$ (2)

where the second is the regular term, ***w*** is the normal vector of the classified hyperplane (Eq. 2), *C* is a constant, and $\xi_{i}$ is the relaxation factor. Here, we introduce the kernel function that can map the sample from the original space to a higher dimensional idiosyncratic space, making the sample linearly differentiable in the new space. The best classification in this study is the Gaussian kernel function with the expression of

$$\kappa \left(x_{i},x_{j}\right)=\exp\left(-\frac{{\left\|x_{i}-x_{j}\right\|}^{2}}{2\sigma^{2}}\right),$$ (3)

where *x*_*i*_ and *x*_*j*_ denote the input categorical feature variables and σ is the width (Eq. 3) of the Gaussian kernel. In this way, a classifier (Figure 1A) for liver pathological states was built based on the SVM method and Python programming language. In each liver tissue sample, 70% of the data is allocated to the training group, while the remaining 30% is assigned to the testing group.

### Ethical approval

It has been confirmed that the experimental data collection complied with relevant institutional, national, and international guidelines and legislation with permission from the administration committee of experimental animals of The Second Affiliated Hospital of Xi’an Jiaotong University, China. All methods reported follow ARRIVE guidelines.

## Results and discussion

### Assessment of classification accuracy for viscoelastic variables

#### The classification of healthy and diseased liver tissues with a single mechanical marker

After building the prediction classifier to output liver pathological states, we evaluated the accuracy of each viscoelastic variable based on the classifier for healthy and diseased livers. In the assessment of the viscoelastic variable of healthy and diseased livers, 70% of the data were treated as the training group and 30% of the data as the test group. The status variables for healthy and diseased liver tissues have been set as 0 and 2, respectively. We first analyze the correlation between the parameters through Pearson’s correlation coefficient. Pearson’s correlation coefficient was calculated by the following formula:

$$r=\frac{\sum \left(x-\bar{x}\right)\left(y-\bar{y}\right)}{\sqrt{\sum {\left(x-\bar{x}\right)}^{2}\sum {\left(y-\bar{y}\right)}^{2}}}$$ (4)

The Pearson correlation coefficient (Eq. 4) of each variable with others is shown in Figure 1B. It indicates that the status of the liver tissue exhibits the highest correlation with the power-law exponent at small time scales $\alpha_{L}$ , followed by the elastic modulus ( $E_{sum}$ , $E_{3}$ , $E_{2}$ , $E_{1}$ ) and transition frequency ( $f_{T}$ ), while the viscosity and power-law exponent at large time scales $\alpha_{R}$ exhibit a minimal correlation with liver status. Moreover, it is noteworthy that the correlation between the elastic moduli is extremely high, suggesting that the increase in elastic moduli during liver lesions is all-encompassing and not limited to the cytoplasm or cytoskeleton. There was a significant positive correlation between the liver tissue lesion and the stiffness, which was consistent with many experiments (Yin et al., 2007; Patel et al., 2015). Different from previous experiments (Wang et al., 2021), we introduced several viscoelastic mechanical variables as classification features and obtained precise correlations between them and liver status. Overall, there is a clear perception that a higher value of $\alpha_{L}$ and a lower value of $E_{sum}$ indicate a lower incidence of liver lesions. In addition, other variables, such as viscosity ( η ), relaxation time ( τ ), and transition frequency ( $f_{T}$ ), showed a relatively low correlation with the status of liver tissue, however, they still have an appreciable degree of accuracy (85%). As shown in Figure 1C, the elastic modulus $E_{sum}$ and $E_{1}$ have the highest accuracy, followed by $\alpha_{L}$ , and meanwhile, the accuracy of the transition frequency reaches more than 0.9. In contrast, the accuracy of $\alpha_{R}$ is extremely low, which is inextricably intertwined with the self-similar hierarchical properties of liver tissues due to its power-law exponent being concentrated around 0.2 at longer time scales. In the third-level hierarchical model, the power-law exponent tends to be constant as the stiffness increases, and thus, the increase in elastic modulus has minimal effect on $\alpha_{R}$ in this case.

#### The classification of healthy and diseased liver tissues with multiple mechanical markers

**FIGURE 2:**
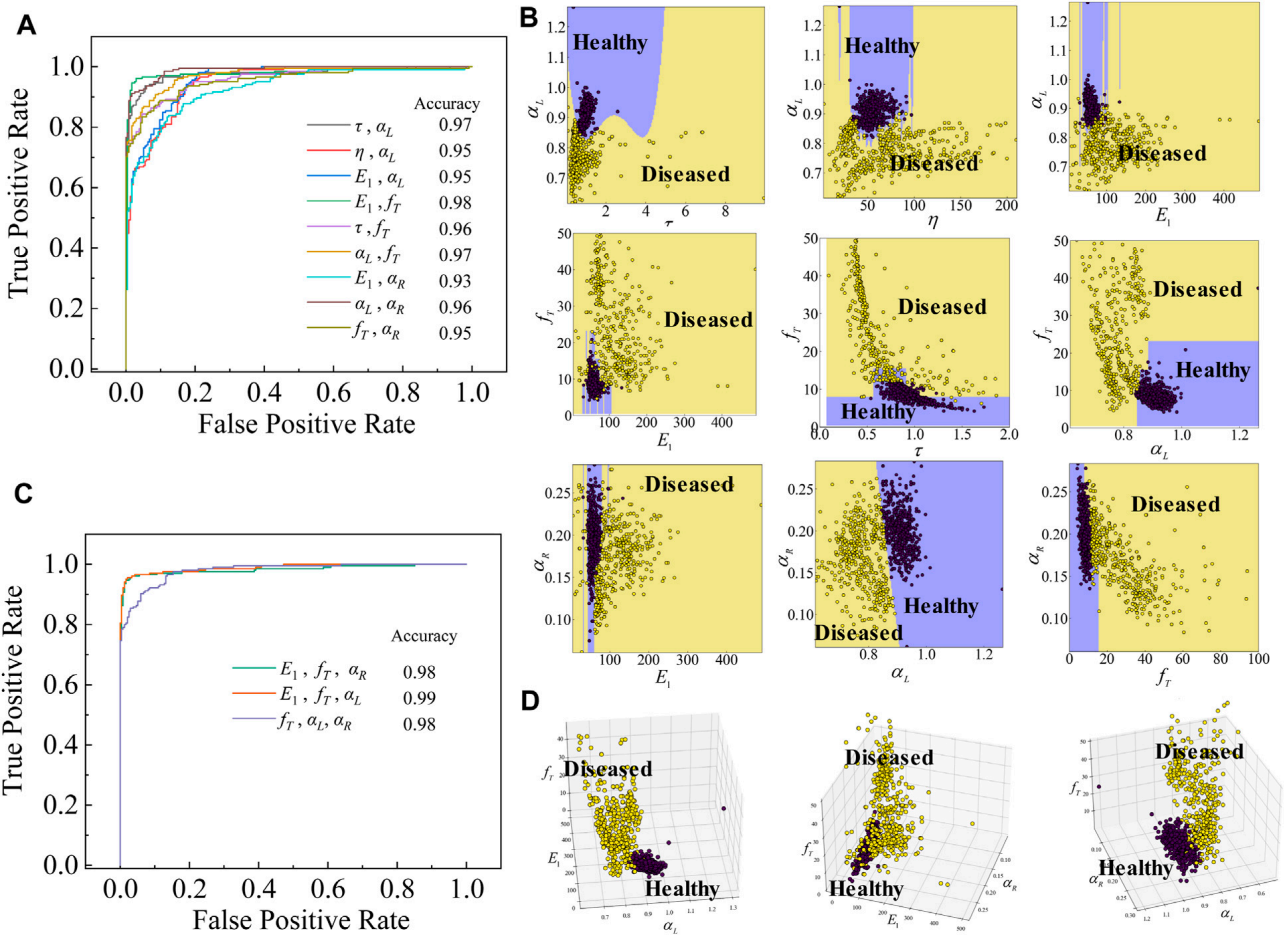
Multiple dynamical mechanical markers performance assessment on the test data group of healthy and diseased livers. **(A)** ROCs of the classifier with two viscoelastic variables as marker input. **(B)** Data points for healthy and diseased livers have their regions when double viscoelastic variables are used as marker input. **(C)** ROCs of the classifier with three viscoelastic variables as marker input. **(D)** When the three viscoelastic variables were used as classification marker input, the data point aggregation feature was more pronounced for healthy and diseased livers compared to that of the two variables used.

As the viscoelastic properties of liver tissues differ substantially between healthy and diseased states, high classification accuracy can be obtained using a single viscoelastic variable (such as $E_{sum}$ and $\alpha_{L}$ ). However, for the early stage of liver fibrosis, the single mechanical property does not change significantly compared to healthy tissues, and at this point, adding variables as classification features contribute greatly to the classification accuracy. Using a combination of viscoelastic mechanical variables without high accuracy as the input marker of the classifier, the classification accuracy could reach a higher level. As shown in Figure 2A, the classification accuracy of the classifier with double variables as classification input markers all reached over 0.93. Based on machine learning, the implied relationships between certain viscoelastic variables could be exploited to improve the accuracy of liver status classification. When two mechanical variables were used to classify healthy and diseased livers, they showed distinct areas of aggregation (Figure 2B). When the number of variables was increased to three, the accuracy of liver classification was further improved (Figure 2C), which benefited from the more pronounced aggregation characteristics of liver status (Figure 2D) at multivariate. Here, the combination of three variables without high accuracy as a single marker input brings the classification accuracy to a higher level, even exceeding 0.98, which is a great improvement compared to that of a single input variable (Figure 1C). We tested nine different combinations of two variables and three different combinations of three variables as input markers of the classifier. Overall, the classification accuracy has reached the best performance when using three viscoelastic variables as marker input, and its classification accuracy even reaches 0.98. This demonstrates that the combination of viscoelastic mechanical variables can capture more salient liver states, enabling a more accurate pathological state prediction of liver tissues, by learning them directly from the implicit connections of the viscoelastic mechanical variables.

### An interpretable predictive model for the liver pathological states

#### Adding diseased tissues after drug treatment to the classifier

**FIGURE 3:**
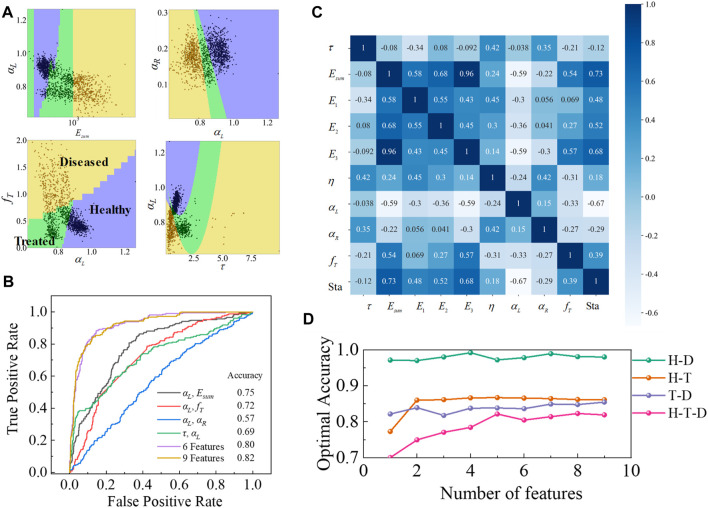
Multiple mechanical markers performance assessment on the test data group of healthy, diseased, and MSCs-treated fibrotic livers. **(A)** Data points for healthy, diseased, and MSCs-treated fibrotic livers have their regions when the two variables are used as classification marker inputs. **(B)** ROCs of the classifier with 2, 6, and 9 viscoelastic variables as marker input. **(C)** The heat map of the Pearson correlation coefficient of viscoelastic variables of healthy, diseased, and MSCs-treated fibrotic liver tissues. **(D)** Optimal accuracy was obtained when different numbers of variables were used for different liver classifications. Here, H, D, and T represent healthy, diseased, and MSCs-treated fibrotic liver tissues, respectively.

Having enabled the classification of healthy and diseased livers, we next expanded the machine learning framework to investigate liver tissues after drug treatment (with the status variable of 1). We trained the prediction classifier and measured the accuracy by combining four different combinations of two viscoelastic variables as input markers to the prediction classifier. For each dataset, we split the data by liver status (healthy, diseased, and MSCs-treated fibrotic) and computed separate confusion matrices to ensure that there is no systematic bias in the predictions. Overall, the best-performing combination is the one combing the elastic modulus $E_{sum}$ and the power-law exponent $\alpha_{L}$ , followed by the power-law exponent $\alpha_{L}$ and transition frequency $f_{T}$ , which had relatively few data points in the junction regions of three liver tissues (Figure 3A). The increase in the number of viscoelastic mechanical variables to the classifier led to an increase in the classification accuracy (Figure 3B). With the introduction of the dataset of diseased livers after drug treatment, there were essentially no apparent changes in the Pearson correlation coefficient (Figure 3C) between the liver status and the individual viscoelastic variables, compared with the dataset of healthy and diseased livers (Figure 1B).

As shown in Refs. (Ziol et al., 2005; Yin et al., 2007), the elastic stiffness of healthy and diseased livers show significant differences. Since many viscoelastic parameters exhibit a strong correlation with the elastic stiffness of tissues, a single viscoelastic variable could achieve a sufficiently high classification accuracy. Consequently, when classifying healthy and diseased livers, we mainly investigated the cases of 2 and 3 variables as the marker input. With the introduction of MSCs-treated fibrotic livers, we used cross-validation to obtain optimal classification accuracy by feature elimination for different numbers of mechanical variables. We implemented a k-fold cross validation with *k* = 10, where the training set was divided into 10 subsamples and one subsample was reserved for model validation, while the remaining nine subsamples were utilized for training. During cross-validation, each subset is iteratively utilized as a test set once, while the remaining k-1 folds are employed as a training set to train the model and assess its performance on each fold. Subsequently, the results from all k evaluations are averaged to derive the final evaluation of the model’s performance. As illustrated in Figure 3D, for the classification of healthy and diseased livers, the optimal classification accuracy is almost independent of the number of input markers, since $E_{sum}$ achieves an accuracy of 0.97 by itself as the single input marker. The introduction of liver tissue data after drug treatment led to a significant trend of increasing the optimal accuracy with the number of input mechanical markers, especially when three liver tissues were mixed for classification accuracy exploration. A comparison of the classification accuracy data of several groups shows that the classification accuracy has reached the optimal value, when the maker number is around 5, after which the increase of markers has little effect on the improvement of accuracy. The viscoelastic variables corresponding to the optimal accuracy of five markers are $E_{sum}$ , $E_{2}$ , $E_{3}$ , $\alpha_{L}$ , and $f_{T}$ , which were also the top five viscoelastic variables in terms of accuracy of classification of healthy and diseased livers as a single marker input in the classifier. Compared to the case when only the elastic modulus $E_{sum}$ was used as a marker, the addition of viscoelastic properties such as the power-law exponent $\alpha_{L}$ and transition frequency $f_{T}$ remarkably improved the classification accuracy of the three liver tissues from 0.7 to 0.82, which is far more significant than the improvement in accuracy in the classification of the two liver tissues. The mean values of elastic modulus ( $E_{sum}$ ) of MSCs-treated fibrotic liver tissues (681.4 MPa) were not significantly different from that of healthy liver tissues (456.1 MPa). The relatively small differences between the elastic moduli of healthy liver tissues and MSCs-treated fibrotic lives suggests the therapeutic efficacy of MSCs in ameliorating liver fibrosis. For diseased liver tissue, the elastic modulus is approximately five times or more than that of healthy tissue (Ziol et al., 2005), which indicates that the elastic modulus may serve as a biomechanical marker for assessing liver fibrosis. When multiple variables were used as input markers, the classification accuracy reached 0.87, indicating that using multiple makers can classify liver tissues in different states by the implied relationships between viscoelastic variables. Therefore, the effect of using all viscoelastic variables as input markers for liver tissue classification will gradually show up with the increasing number of liver tissue statuses such as the grading of liver fibrosis, and eliminates the process of mechanical marker screening.

#### Feature ablation studies

**FIGURE 4:**
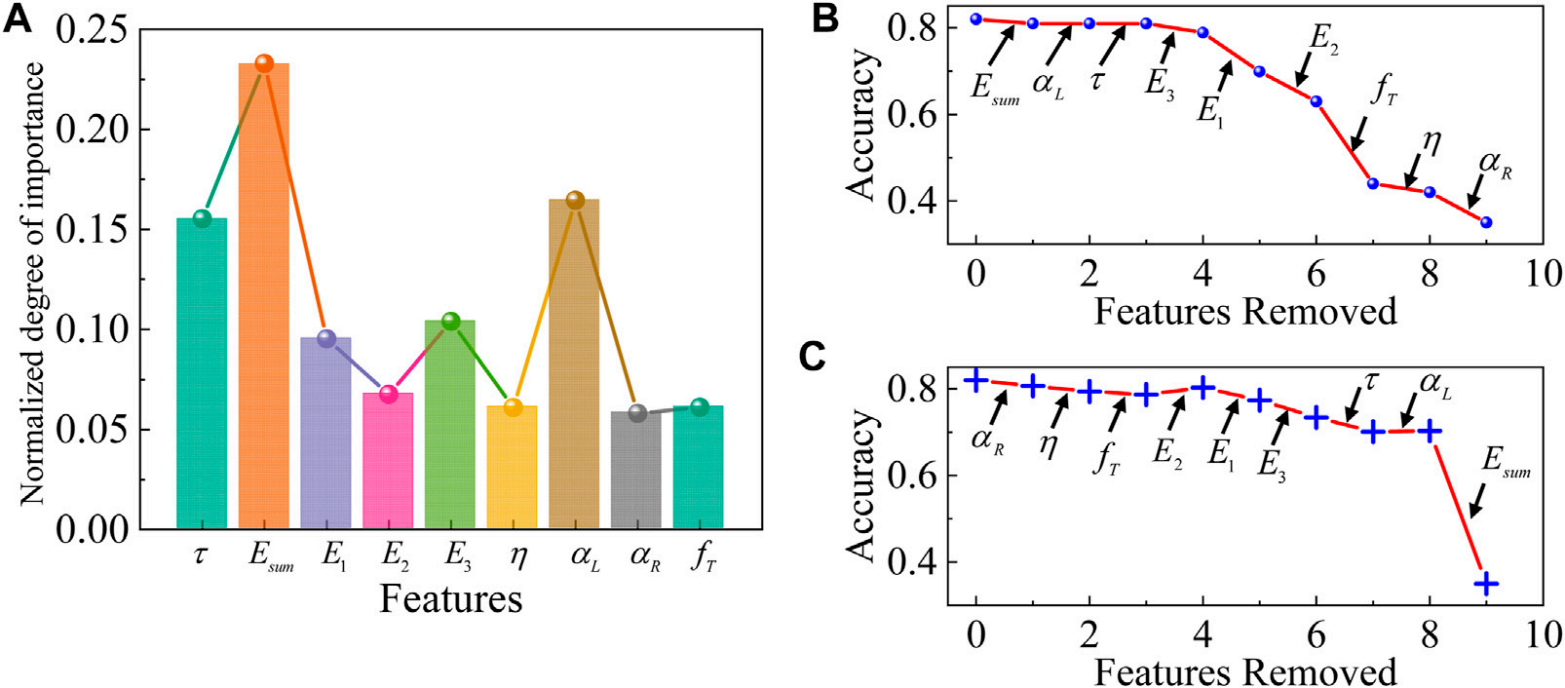
Feature ablation studies of the classifier with healthy, diseased, and MSCs-treated fibrotic liver tissues together. **(A)** The normalized importance of each feature. Feature ablation **(B)** from high to low importance and **(C)** from low to high importance. Feature ablation demonstrates the role of each principal viscoelastic variable in the prediction of the classifier. Each arrow indicates the cumulative replacement of a given principal viscoelastic variable with Gaussian noise.

In this study, we used nine viscoelastic mechanical variables as input markers in the classification of liver pathological states, but in fact, not only do the viscoelastic properties of liver tissues change when lesions occur, but also other properties such as plasticity, component protein characteristics, and image characteristics. Combining viscoelastic properties with these characteristics will greatly improve the quality of liver lesion diagnosis, but will also result in a considerable computational requirement, therefore, a reasonable selection of markers is crucial to improve the efficiency of liver diagnosis. To determine the minimal information required for liver state prediction, we first determined the importance (Figure 4A) ranking of the viscoelastic features of the tissue using Support Vector Machine Recursive Feature Elimination (SVM-RFE). Subsequently, recursive feature ablation is conducted via cross-validation to determine the optimal number of features. Then, we systematically removed individual principal viscoelastic variables (replacing them with Gaussian noise) and calculated the performance of the classifier after the removal of each viscoelastic variable. Through multiple iterations, we found that a single mechanical marker ( $\alpha_{R}$ ) could be used to predict liver tissues with 42% accuracy—apparently higher than random chance assuming the same probability of selecting a liver status (33.33%, Figure 4B). The elimination of $E_{sum}$ in the positive-order feature ablation (Figure 4C) does not generate a significant decrease in accuracy, but the elimination of $E_{1}$ and $E_{2}$ has a significant effect on accuracy, which results from the high correlation between the elastic moduli, and a precipitous drop in accuracy when $E_{sum}$ is finally eliminated in the inverted-order feature ablation. Feature ablation studies demonstrated that $E_{sum}$ was the viscoelastic variable that contributed most to prediction accuracy. Feature ablation studies can determine the importance of input biomarkers and filter out invalid feature variables, thus improving classifier efficiency in high-dimensional data classification and liver diagnosis.

#### A method for evaluating drug treatment effects

Having established the SVM-based classifier that can classify livers in an interpretable manner, we sought to define a new approach to drug-based screening using a predictive classifier. To this end, we tested the classification accuracy of liver tissues after drug treatment against healthy and diseased livers to determine the effect of the indicated drug on liver states. The accuracy of classification of treated livers with healthy and diseased livers reached 0.86 and 0.85 (Figure 3D), respectively. The MSCs-treated fibrotic liver tissue was clearly classified from diseased liver tissues and there is a tendency for the elastic modulus to be greatly reduced, which indicates that the drug treatment has freed them from the diseased state. The accurate classification of healthy liver tissues also indicates that the drug treatment has not completely restored them to a healthy state. Overall, drug treatment allows the liver tissue to recover from the disease to healthy state. The accuracy of the classification of liver tissue after drug treatment compared to healthy and diseased liver tissues allows a clear determination of the effect of drug treatment and the need for continued drug use. When the classification accuracy of the MSCs-treated fibrotic liver tissues with healthy liver tissues is reduced to 0.33 and the classification accuracy with diseased tissue reaches a high level, we can assume that the liver tissue has recovered to its original state under drug treatment, which is, of course, the ideal situation. As an example, our method can be used to determine the effect of a drug by screening the recovery of viscoelastic properties induced by the drug. We can further analyze the changes in the main viscoelastic biomarkers to determine the target of the action of the drug. In addition, in the process of liver fibrosis, there exists a grading of its lesion degree. At this time, our three-class classifier can be further extended to achieve accurate grading of liver fibrosis and provide the basis for subsequent treatment.

## Discussions

Machine learning is now a powerful tool for medical diagnosis. Although many machine learning diagnostic models are mainly based on the recognition of image technology, they lack suitable mechanical markers, which makes them rarely used to identify tissue lesions in similar states or less severe lesions. Here, we analyzed the creep responses of liver tissues by a self-similar hierarchical model and obtained the viscoelastic properties of liver tissues in different states. Then, we built an SVM-based machine learning classifier with viscoelastic properties as input mechanical markers. Remarkably, our SVM classifier successfully identifies elastic modulus $E_{sum}$ and power-law exponent $\alpha_{L}$ as the strongest predictors of liver tissue status. Furthermore, the addition of many viscoelastic variables makes the accuracy of this classifier greatly improved compared to the case where only a single variable is used. An extension of this work would be the use of this classifier for the quantitative assessment of drug treatment effects. The classification accuracy of liver tissue after drug treatment with healthy and diseased liver tissues can be obtained towards the classifier, and a lower classification accuracy with healthy tissue and a higher classification accuracy with diseased tissue indicated a better recovery effect of the drug. Furthermore, it is also feasible to combine multiple different states of liver tissue for classification, which provides a new strategy for grading liver fibrosis. For such cases, we also give screening methods based on feature ablation for inputting biomarkers at high dimensional data. We have shown that a novel classifier, based on the learned model, can predict the pathological states of liver tissue based on the implicit relationship of viscoelastic biomarkers. Once trained, this fully automated classifier can distinguish between normal, diseased, and MSCs-treated fibrotic liver tissue without any further human intervention, paving the way for drug screening and development. Currently, creep testing relies on liver tissue sections, which, despite their clinical utility, pose significant limitations. Biopsy-based procurement of liver tissue sections for clinical purposes is invasive, causing damage and discomfort. AFM measurements also fall slightly short in facilitating large-scale lesion diagnosis. However, this study presents a novel approach for characterizing liver tissue lesion progression. There are two main advantages of our proposed method in relation to existing methodologies. One is the richness of viscoelastic mechanical markers, which is conducive to improving the reliability of diagnosis. The second is the quantization of viscoelastic mechanical markers, which is conducive to improving the accuracy of diagnosis compared with the qualitative judgment of imaging. In future work, improving the culture conditions to achieve precise staging of liver lesions in mice will be the focus. Subsequently, further validation of the method proposed in this study will then be carried out, based on the improved staging of liver biopsies using fibrosis scoring systems. With the advancement of non-invasive detection techniques for liver tissues, our proposed viscoelastic mechanics markers and machine learning-based diagnostic method offer valuable insights for diagnosing liver tissue lesion progression.

## Data Availability

The original contributions presented in the study are included in the article/Supplementary Material, further inquiries can be directed to the corresponding authors.
